# Supplementary material for: Identification of Genes Associated with Gut Microbiota Metabolites in Type 2 Diabetes Mellitus Complicated by Acute Cerebral Infarction Based on Bioinformatics and Mendelian Randomization
Source: Biomedicines. 2026 Oct 17;14(10):2371. doi: 10.3390/biomedicines14102371 (PMC13702449; doi:10.3390/biomedicines14102371)
Supplement: Supplementary file 1 [file biomedicines-14-02371-s001.zip › Figure S1/family.Verrucomicrobiaceae.id.4036 ACI.pdf]

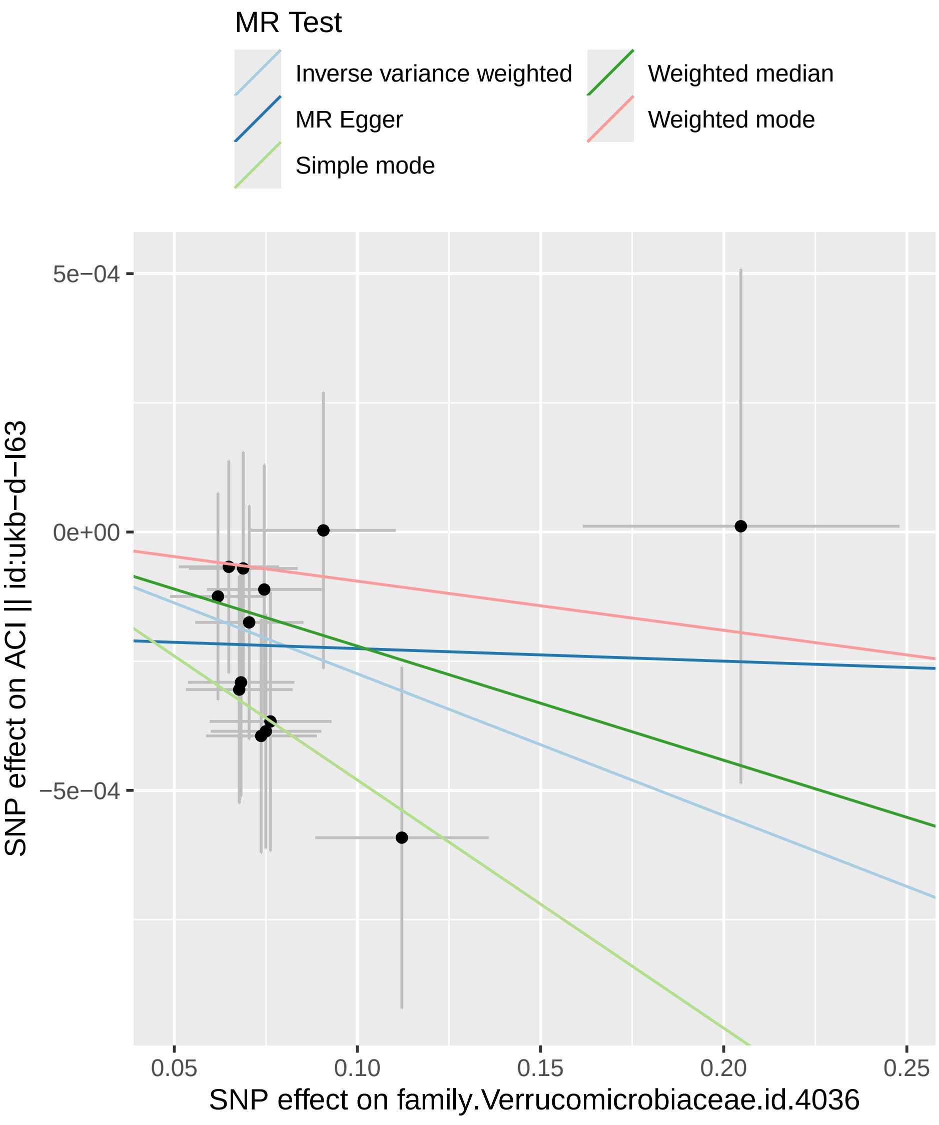

MR Test
Inverse variance weighted
MR Egger
Simple mode
Weighted median
Weighted mode
5e−04
0e+00
−5e−04
0.05
0.10
0.15
0.20
0.25
SNP effect on ACI || id:ukb−d−I63
SNP effect on family.Verrucomicrobiaceae.id.4036
